# Life stress and suicidal ideation in Australian men – cross-sectional analysis of the Australian longitudinal study on male health baseline data

**DOI:** 10.1186/s12889-016-3702-9

**Published:** 2016-10-31

**Authors:** Dianne Currier, Matthew J. Spittal, George Patton, Jane Pirkis

**Affiliations:** 1 Centre for Epidemiology and Biostatistics, The University of Melbourne, Melbourne, 3010 Australia; 2 Centre for Mental Health, The University of Melbourne, Melbourne, 3010 Australia; 3 Centre for Adolescent Health, Murdoch Childrens Research Institute, Parkville, 3052 Australia

## Abstract

**Background:**

Suicide is a leading cause of death in Australian males aged 18 to 55. Non-fatal suicidal behaviours and thoughts are indicators of increased risk for future suicide. Suicidal behaviour is complex and multi-determined. Research supports the involvement of stressful life events in suicide and suicidal behaviour, however the evidence regarding suicidal thoughts is less developed. This study investigates stressful life events in relation to suicidal ideation in a large cohort of adult males recruited into Ten to Men, the Australian Longitudinal Study on Male Health.

**Methods:**

Baseline data from a national cohort of 13, 884 males aged 18–55 years on suicidal behaviour, psychiatric disorder and life events was used. Multivariable logistic regressions were conducted with current suicidal ideation as the outcome and 12 month life events, 12 month depression, anxiety and harmful/hazardous alcohol use, and socio-demographics as covariates. Further logistic regression models investigated the relative risk of life stress alone, depression/alcohol/anxiety alone and co-occurring life stress and depression/alcohol/anxiety.

**Results:**

In multivariable models there was an independent contribution to suicidal ideation for six of 24 life events (ORs 1.27–1.95), 12 month depression (OR 4.49) harmful alcohol use (OR 1.38) and anxiety disorders (OR 1.27). Life events co-occurring with depression (OR 10.3) was higher risk than either alone (depression OR 6.6; life stress OR 2.6). There was a lesser effect for co-occurrence in the anxiety and harmful alcohol use models.

**Conclusion:**

Life events appear to be related to suicidal ideation independent of depression, anxiety and harmful alcohol use in adult males, however if life events occur in the context of depression that risk is substantially increased.

## Background

Suicide is a leading cause of premature mortality in Australian males and in 2013 accounted for 63,433 years of potential life lost [1]. In that year, it was the leading cause of death in Australian males aged 15–45 and the third leading cause in males aged 45–55 [1]. Suicidal thoughts and non-fatal suicidal behaviours occur more frequently than suicide, cause considerable morbidity and are a strong predictor of risk for future suicide death. A broad range of risk factors for suicide and suicidal thoughts and behaviours have been identified in clinical and population studies (see Nock [2] for a review). Explanatory models have been developed to describe how these risk factors interact and the putative causal pathways for suicidal thoughts and behaviours. In the stress-diathesis model, the diathesis is understood as a predisposition to think about suicide or engage in suicidal behaviour when exposed to stressors [3]. The diathesis includes predisposing factors such as mental illness, alcohol and substance use, impaired decision making, traits such as aggression, hopelessness, and impulsivity, and dysfunction in key neurotransmitter systems including stress-response systems (see van Heeringen [4] for a review). The stress domain includes stressful life events and other state-based factors such as an episode of physical illness.

A role for life stressors in suicidal behaviours is supported by a large literature. A recent systematic review found consistent reports of associations between negative life events and suicide ideation, attempt and suicide, although the evidence was least robust for ideation [5]. While life stressors are an obvious potential trigger for a suicidal crisis, many such stressors are widely occurring and in most individuals do not result in suicidal thoughts or behaviours, indicating that other factors (i.e., elements of the diathesis) may also be involved. Understanding the role of life stressors in suicidal ideation in males, including their interaction with the diathesis for suicidal behaviour, is important for suicide prevention as it may identify potential points for early intervention before individuals escalate to more serious suicidal behaviours.

Ten to Men is a population-based study of the health of a large cohort of males aged 10–55 at recruitment. At baseline, data were collected on suicidal thoughts and behaviours, and on constructs relevant to both stress (i.e., life events) and diathesis (i.e., major psychiatric disorders) domains. This study uses the baseline data to examine the relationship of life stressors and psychiatric disorders in the past 12 months to current suicidal ideation. It assesses the salience of different stressful life events and examines the relative contribution of life events and psychiatric disorders with respect to suicidal ideation in males aged 18–55.

## Methods

### Sample

Ten to Men recruited 15,988 males aged 10–55 years from across Australia in 2013/14 using a stratified clustered random sampling strategy. Males were included if they were resident in a private dwelling, were an Australian citizen or permanent resident and had sufficient proficiency in English to complete the questionnaire/interview. Sampling, recruitment and data collection methods are described elsewhere [6]. For this analysis males 18 years and older were included (*n* = 13,884) as they are exposed to a different and wider set of life stressors than younger males.

### Measures

Lifetime suicidal thoughts and behaviours were ascertained by three standard questions: *Have you ever seriously thought about killing yourself?; Have you ever made a plan about how you would kill yourself?*; *Have you ever tried to kill yourself?*. The item *‘Thoughts that you would be better off dead or of hurting yourself in some way’* from the Patient Health Questionnaire (PHQ9) was used to capture suicidal ideation in the past 2 weeks [7], which we deemed to be current suicidal ideation. Respondents indicated the frequency of such thoughts in the past 2 weeks as ‘not at all’, ‘several days’, ‘more than half the days’, and ‘nearly every day’. Self-report psychiatric illness was ascertained by two questions: *‘Has a doctor or other health professional ever told you that you had this condition?’* for lifetime prevalence, and ‘*Have you been treated for or had any symptoms of this condition in the past 12 months?’* for 12 month prevalence. Mental health conditions available for endorsement were depression, post-traumatic stress disorder (PTSD), other anxiety disorders, and schizophrenia. This format for capturing self-reported long-term conditions was based on the Australian Health Survey [8]. Alcohol use was assessed using the Alcohol Use Disorders Identification Test (AUDIT) in which harmful or hazardous alcohol use is defined as an alcohol dependence score of 8 or more [9]. Life events in the prior 12 months were ascertained using a life events checklist comprising 24 items. The majority of the items (19) had been used in the Australian Longitudinal Study of Women’s Health surveys, based on their adaption of the Social Readjustment Scale [10]. Modifications included two instances of combining multiple items into one (*serious personal injury, illness or surgery*; *break-up of a serious relationship/divorce/separation)*. Two items were modified to be more general (*being pushed, grabbed, shoved, kicked or hit* modified to *victim of physical violence*, *being forced to take part in unwanted sexual activity* modified to *being a victim of sexual assault*) one item broadened in scope (*loss of a job* to *loss of a job or looking for work unsuccessfully for a long time*) and one revised to be male specific (*birth of your first child* to *becoming a father for the first time)*. Five additional items were included, four to capture important transition points: *starting your first job, retirement, bankruptcy (personal or business), buying a house, becoming a carer for someone*, and one from the Social Readjustment Scale (*serious illness, injury or assault to a close relative/friend)* [11]. There was no rating of the severity of stress associated with the event.

Participant socio-economic status was based on the Australian Bureau of Statistics, Socio-Economic Indexes for Areas - Index of Relative Socio-Economic Disadvantage (SEIFA-IRSD). This is general socio-economic index produced from census data summarising a range of indicators of economic and social disadvantage [12]. Lower IRSD scores indicate greater disadvantage.

### Statistical analyses

Frequencies for clinical factors and suicidal behaviour were produced using data weighted to the general population age and regional distribution (for details on design and use of sample weights see Spittal et al. [13]). All 24 life events variables were entered into a logistic regression model with current suicidal ideation as the outcome variable, and socio-demographics (10 year age group, marital status, socio-economic status), and clinical factors (12 month depression, 12 month anxiety and 12 month harmful/hazardous alcohol use) as covariates. This regression did not adjust for the sample design or for sample weighting. Life events that were not significant at the *p* < 0.05 level were removed and the model was re-estimated.

To examine stress/diathesis effects we constructed variables representing combinations of stressors and putative elements of the diathesis (12 month depression, 12 month anxiety, 12 month harmful/hazardous alcohol use). We constructed three variables representing each diathesis element. Each variable could take one of four values and had the form: (1) no stressors present and no diathesis present; (2) stressors present but no diathesis present; (3) no stressors present but diathesis present; and (4) both stressors and diathesis present. We defined the stressor variable as the participant reporting “yes” on any of the life events that remained significant in the multivariable model described above. We then conducted separate logistic regressions for stressor/depression, stressor/anxiety and stressor/alcohol use with current suicidal ideation as the outcome variable (also without adjustment for sample design or weighting). We also included the socio-demographic variables described above and the two other clinical variables not included in that model’s ‘diathesis’ as covariates.

## Results

### Prevalence of suicidal behaviour, depression, anxiety and alcohol use

Table 1 gives the weighted population counts and frequencies for suicidal ideation, suicide plans, suicide attempts, depression, anxiety and alcohol use disorder. High levels of psychiatric disorders and suicidal behaviour were reported, with one in five males aged 18 and 55 years reporting lifetime depression, a similar proportion (18 %) reporting lifetime suicidal ideation, and 9.5 % reporting experiencing suicidal ideation in the past 2 weeks. More than one in twenty reported having made a suicide attempt in their lifetime.

**Table 1:** Frequency of lifetime suicidal behaviour, depression, anxiety, 12 month depression, anxiety and harmful/hazardous alcohol use and current suicidal ideation (weighted)

Lifetime	*N*	%
Suicidal ideation	958,607	18.6
Suicide plan	537,972	10.5
Suicide attempt	278,375	5.4
Depression	1,012,958	20.0
Anxiety	687,566	13.4
12 Month		
Depression	651,880	12.8
Anxiety	448,174	8.8
Alcohol harmful/hazardous Use	1,718,560	33.6
Current (2 Week)		
Suicidal ideation	487,118	9.5

### Suicidal ideation and life events

Table 2 gives unweighted frequencies of all 24 life events assessed.

**Table 2:** Frequency of life events (unweighted)

Life Event	Number	%
Serious personal injury, illness or surgery	1,997	14.4
A serious illness, injury or assault to a close relative/friend	2,977	21.4
Break-up of a serious relationship/divorce/separation	991	7.1
Getting Married (or starting to live with someone)	894	6.4
Infidelity of partner or spouse	297	2.1
Becoming a father for the first time	545	3.9
Serious conflict with family member	1,577	11.4
Death of partner, spouse or close family member	1,909	13.7
Loss of a child (e.g., death, stillbirth, miscarriage)	311	2.2
Left home for the first time	362	2.6
Child or family member left home	835	6.0
Starting your first job	347	2.5
Difficulty finding a job	2,283	16.4
Loss of job, looking for work unsuccessfully for a long time	1,531	11.0
Retirement	71	0.5
Bankruptcy (personal or business)	128	0.9
Natural disaster (fire, flood, drought, earthquake, etc.)	464	3.3
Major loss or damage to personal property	441	3.2
Being a victim of sexual assault	33	0.2
Victim of physical violence	325	2.3
Legal troubles or court case	813	5.9
Buying a house	1322	9.5
Moving house	2935	21.9
Becoming a carer for someone	422	3.0

In multivariable analysis, six life event variables remained significantly associated with current suicidal ideation (Table 3). These were serious family conflict, difficulty finding a job, legal troubles, major loss of property, break-up of a relationship and serious personal injury (odds ratios [ORs] between 1.27 – 1.95). Among the socio-demographic covariates, age had no effect on likelihood of suicidal ideation, and being married or in a de-facto relationship and residing in areas of least socioeconomic disadvantage were protective. Among the clinical factors, those with self-reported depression in the past 12 months had four times the odds of experiencing suicide ideation (OR 4.49 95 % CI: 3.74,5.40) compared to those without depression. Past 12 month anxiety was associated with a more modest increase in the odds of experiencing suicidal ideation (OR 1.27 95 % CI: 1.02,1.57) as was past 12 month harmful/hazardous alcohol use (OR 1.38, 95 % CI:1.20,1.59).

**Table 3:** Correlates of current week suicidal ideation (unweighted)

Correlate	OR	95 % CI	*p*-value
Socio-demographic factors			
Age			
(base) 18–29	1.00		
30–39	0.95	0.77,1.17	0.613
40–49	1.12	0.92,1.38	0.263
50–55	1.11	0.88,1.40	0.370
*Married/* *de facto*	*0.69*	*0.58, 0.81*	*0.000*
Socioeconomic disadvantage^a^			
(base) 1st quintile	1.00		
2nd quintile	1.09	0.87,1.35	0.456
3rd quintile	0.90	0.73,1.11	0.330
4th quintile	0.97	0.78,1.21	0.819
*5th quintile*	*0.73*	*0.58,0.93*	*0.010*
Clinical factors			
*Depression (12 month)*	*4.49*	*3.74,5.40*	*0.000*
*Anxiety (12 month)*	*1.27*	*1.02,1.57*	*0.025*
*Alcohol Harmful/Hazardous Use*	*1.38*	*1.20,1.59*	*0.000*
Life Events (12 month)			
*Serious personal injury*	*1.27*	*1.07,1.51*	*0.006*
*Relationship end*	*1.46*	*1.18,1.80*	*0.001*
*Family conflict*	*1.95*	*1.64,2.32*	*0.000*
*Difficulty finding work*	*1.95*	*1.65,2.39*	*0.000*
*Legal troubles*	*1.27*	*1.00,1.60*	*0.046*
*Loss of personal property*	*1.66*	*1.23,2.23*	*0.001*

^a^Australian Bureau of Statistics, Socio-Economic Indexes for Areas - Index of Relative Socio-Economic Disadvantage

### Stress-diathesis

Table 4 presents results of the analysis of additive effects of life stress and clinical elements potentially related to the diathesis for suicidal behaviour. For depression and life events, a strong additive effect was found, whereby co-occurrence of depression and a stressful life event in the past 12 months increased odds of experiencing suicidal ideation considerably (OR 10.3), compared to depression alone (OR 6.6) and life-event alone (OR 2.6). A smaller additive effect was found for both anxiety disorder and harmful/hazardous alcohol use and life events with higher odds for co-occurring disorder and life events compared to the disorder alone or life events alone.

**Table 4:** Stress-diathesis Regression models: Current suicidal ideation as the outcome variable

MODEL	Adjusted OR	95 % CI	*p* value
DEPRESSION^a^			
No stressors and no depression	1		<0.001
Stressors and no depression	2.6	2.2; 3.1	
No stressors and depression	6.6	5.0; 8.6	
Stressors and depression	10.3	8.2; 12.8	
ANXIETY ^b^			
No stressors and no anxiety	1		<0.0.001
Stressors and no anxiety	2.4	1.9; 2.4	
No stressors and anxiety	1.7	1.2; 2.4	
Stressors and anxiety	3.0	2.3; 3.9	
ALCOHOL USE ^c^			
No stressors and no heavy alcohol use	1		<0.0.001
Stressors and no heavy alcohol use	2.4	1.9; 2.9	
No stressors and heavy alcohol use	1.5	1.2; 1.9	
Stressors and heavy alcohol use	3.2	2.6; 3.9	

^a^adjusted for age, marital status, SEIFA IRSD, anxiety, alcohol use

^b^adjusted for age, marital status, SEIFA IRSD, depression, alcohol use

^c^adjusted for age, marital status, SEIFA IRSD, anxiety, depression

## Discussion

### Suicidal behaviour

The rates of suicidal thoughts and behaviour in Ten to Men adult respondents are higher than reported in the 2007 National Survey of Mental Health and Wellbeing (NSMHWB), the benchmark population data collection on non-fatal suicidal thoughts and behaviours in adults in Australia. Lifetime suicidal ideation was reported by 18.8 % of Ten to Men adult respondents compared to 11.5 % of males in the NSMHWB, and lifetime suicide attempts were reported at more than twice the rate in Ten to Men adult respondents (5.4 % compared to 2.2 %) [14]. Likewise lifetime and 12 month depression was reported more frequently in the Ten to Men adult cohort (depression: 20 % lifetime and 12.8 % 12 month) than in the NSMHWB (depressive episode: 8.8 % lifetime and 3.1 % 12 month) [14], although anxiety was less frequently reported (Ten to Men: 13.4 % lifetime and 8.8 % 12 month; NSMHWB: 20.4 % lifetime and 10.8 % 12 month) [14]. These differences in rates likely reflect methodological differences including the use of self-report questionnaires in Ten to Men versus face-to-face interview in the NSMHWB and the use of clinical interview to assess depression and anxiety in the NSHWBS compared to a general question about lifetime diagnosis in Ten to Men. While the study design aimed to obtain a representative sample and sample weights adjusting for age and region have been applied to the analysis [13], Ten to Men is not designed as a cross-sectional prevalence study but rather a longitudinal study aimed at investigating the causes of and pathways to health outcomes within individuals. As such, a cohort enriched for suicidal behaviour and psychiatric disorder offers greater scope to investigate the complex causality of suicidal behaviour.

### Life stress and suicidal ideation

In the initial multivariable analysis, elements from both the stressor and diathesis domains increased risk for suicidal ideation. Depression, anxiety and alcohol use, all putative elements of the diathesis for suicidal behaviour, were associated with increased risk for suicidal ideation. Depression in the past 12 months was the most robust predictor with more than twice the effect size of any other risk factor in the model. In the stressor domain six of the twenty-four life events assessed made an independent contribution to risk for suicide ideation. While a smaller effect than depression, these life events contributed to risk in the same magnitude as anxiety and harmful/hazardous alcohol use, suggesting that the stressor domain has an important role and is thus a potential target for intervention in suicide prevention.

The six life events associated with increased odds of suicidal ideation were serious family conflict, break-up of a relationship, difficulty finding a job, legal troubles, major loss of property and serious personal injury. Of these, events in the employment and interpersonal domains had somewhat stronger effects. Liu and Miller in their recent systematic review found some evidence that relative to other types of stressors interpersonal difficulties were more often associated with suicidal thoughts although they did not present data specific to males [5]. In this study, the variable nature of the six significant life events and the modest differences in effect sizes between them makes it difficult to identify particular domains or types of life events that are higher risk than others. Moreover, while certain life events were found to be more strongly associated with increased risk for suicidal thoughts than others, the likely collinearity between life events and the low frequencies of certain events (e.g., loss of a child) may have meant that events which are highly stressful were not retained in the final models. Thus no general conclusions can be drawn about the salience of particular life events over others with respect to risk for suicidal thinking. The variability may also indicate that the type of life event is less important to risk for suicidal ideation than the broader context in which any particular life event takes place, including the presence of elements of the diathesis.

While we cannot elucidate causal pathways in a single wave of data, we can investigate if the co-occurrence of a stressor and elements of the diathesis increases risk for suicidal behaviour. In the case of depression, we found this to be the case. The co-occurrence of life events and depression in the past 12 months resulted in an almost four-fold increase in risk for suicidal ideation over life events with no co-occurring depression, as well as a 35 % increase in the odds of suicidal ideation compared to depression in the absence of life events. While this elevated risk for suicidal ideation associated with co-occurring stressors and depression is consistent with a stress-diathesis model, our analysis also indicates that life events plus depression does not fully encapsulate the stress-diathesis pathway. Independent effects for both life stress and depression persisted in the model and, moreover, the magnitude of increase in ORs associated with co-occurring life stressors and depression did not demonstrate the multiplicative relationship as might be expected if life events plus depression alone constituted the stress-diathesis pathway.

The independent risk associated with life events, depression and, to a lesser degree, anxiety and alcohol use, likely reflects the multi-dimensional nature of both the stressor and diathesis domains. Thus, in the absence of depression other diathesis elements such as deficits in cognitive function, maladaptive stress response, aggression, impulsivity etc. not included in this analysis may be pathways to suicidal ideation in response to life stressors. Likewise, in the stressor domain we assessed only a limited range of life events on the more severe end of the spectrum, and among those in the ‘depression alone’ group reporting suicidal thoughts other life stressors may be occurring. Given the multi-determined nature of suicidal ideation and behaviours it is also likely that there are multiple phenotypes and causal pathways. More complex modelling that includes a broader range of stress and predisposing factors is required to elucidate these. However, this limited analysis of more commonly occurring stressors and psychiatric disorders does indicate that the co-occurrence of life stress and depression substantially increases the risk of suicidal ideation. It also indicates a need for further research to identify predisposing factors in non-depressed males which may increase their vulnerability to suicidal ideation and behaviour when encountering fairly common life stressors in order to identify targets for prevention in this group.

### Limitations

The outcome measure of suicidal ideation was drawn from a composite scale designed to assess depression rather than from a suicide assessment instrument and, as such, may lack specificity. Moreover, given that suicidal ideation is a symptom of depression, individuals who were positive for current depression were more likely to have endorsed current suicidal ideation and 12 month depression. While this may overestimate the magnitude of the association between depression and suicidal ideation, the finding that several life events remained significantly associated with suicidal ideation after controlling for depression indicates the robustness of that association. Other limitations are those found in life event studies using self-report checklists regarding reliability of recall of past life events [15]. While the relatively short recall period (past 12 months) and the serious nature of the life events assessed may reduce recall error in Ten to Men this remains a limitation of the checklist method for capturing life event information. Moreover, as with all cross-sectional studies a single wave of data can only discern correlations. Thus we cannot rule out reverse causality whereby individuals who are currently depressed and suicidal may differentially recall more adverse events compared to those who are not, as has been reported in some studies, but not all [16, 17]. Finally, this study does not include any assessment of early-life stressors. Early life trauma has been associated with suicidal behaviour [18] and a number of putative pathways implicated including neurodevelopmental deficits resulting in impaired stress response later in life and greater vulnerability to psychiatric disorder [19]. This study focussed on proximal life stressors (past 12 months) as putative ‘triggers’ to current suicidal ideation, however it is likely that for some individuals early-life stressors contribute to the association between suicidal thoughts and life events observed here, and that analyses examining both distal and proximal stressors are required to fully elucidate the relationship between life stress and suicidal ideation. Over its longitudinal course, Ten to Men will be better able to track the timing of life stressors and identify causal pathways between proximal and distal life events, psychiatric illness and suicidal behaviour.

## Conclusion

Understanding the pathways and mechanisms through which life events contribute to suicidal behaviour is important to development of prevention strategies. We found that a number of stressful life events were associated with increased risk for suicidal ideation and while this was not accounted for entirely by the co-occurrence of psychiatric disorder if those life events occurred in the context of depression the odds for suicidal ideation increased substantially. The observation of a contribution of a number of life events to suicidal ideation independent of depression, alcohol and anxiety disorders also indicates a need for prevention strategies with a wider focus than treatment of psychiatric illness.
